# School and Teacher Factors That Promote Adolescents’ Bystander Responses to Social Exclusion

**DOI:** 10.3389/fpsyg.2020.581089

**Published:** 2021-01-11

**Authors:** Kelly Lynn Mulvey, Seçil Gönültaş, Greysi Irdam, Ryan G. Carlson, Christine DiStefano, Matthew J. Irvin

**Affiliations:** ^1^Department of Psychology, North Carolina State University, Raleigh, NC, United States; ^2^Department of Educational Studies, University of South Carolina, Columbia, SC, United States

**Keywords:** bystander intervention, peers, discrimination, teachers, school climate, inclusion

## Abstract

Schools may be one important context where adolescents learn and shape the behaviors necessary for promoting global inclusivity in adulthood. Given the importance of bystanders in halting bullying and peer aggression, the focus of this study is on both moral judgments regarding one type of bullying, social exclusion, and factors that are associated with bystander intervention. The study includes 896 adolescents, who were 6th (*N* = 450, *M_*age*_* = 11.73), and 9th (*N* = 446, *M_*age*_* = 14.82) graders, approximately evenly divided by gender. Participants were primarily European–American (63.3%). Results revealed that girls and participants who perceived better relationships between students and teachers were more likely to judge exclusion to be wrong. Further, ethnic minority participants, those who were more anxious about being rejected by their teachers and reported more teacher discrimination were less likely to judge exclusion as wrong. Participants who reported more positive student–teacher relationships, perceptions of a more positive school social environment and more prior experiences of teacher discrimination were more likely to report that they would seek help for the victim. On the other hand, participants who reported being more angry about teacher rejection, experiencing either peer or teacher discrimination, and perceiving they are excluded from opportunities at school were less likely to intervene to come to the aid of a peer who is being excluded. The results document the complex interplay of school and teacher factors in shaping adolescents’ bystander responses to social exclusion. Our findings suggest that positive school climate can promote intentions to intervene. However, findings indicate that adolescents who are marginalized in their school environments, and who report experiences of rejection, exclusion or discrimination are not willing or likely to intervene to prevent others from experiencing exclusion.

## Introduction

Adolescents experience social exclusion and observe others who are excluded (Abrams et al., 2005; Killen and Rutland, 2011). While researchers have often examined social exclusion with the aim of understanding youth experiences of exclusion and exploring their evaluations of and reasoning about exclusion (Rutland and Killen, 2015; Mulvey, 2016), it is imperative to also understand what factors predict inclusive behavior. This is especially important given the negative impacts of exclusion on short- and long-term well-being, academic success and mental health (Buhs et al., 2006). Furthermore, the United Nations, which has placed a strong focus on ensuring global inclusive societies, articulates that social exclusion can manifest in many ways, including rejection from group activities, denial of educational and occupational opportunities, restricted access to social support, and systematic inequality (United Nations., 2016). Finally, the Organization for Economic Co-operation and Development noted that over the past decade, schools have struggled to make progress in both academic and social inclusion (OECD, 2015). Although, as noted above, exclusion can occur in community settings as well (for instance, in informal peer interactions), schools may be one important context where adolescents learn and shape the inclusive behaviors necessary for promoting global inclusivity in adulthood. Thus, we examine adolescents’ bystander behaviors, with attention to school and teacher factors that promote adolescents’ defending behaviors when they observe social exclusion, a type of bullying. In particular, we examine school climate, as key research has documented that school climate can shape feelings of inclusion and belonging (Cemalcilar, 2010), teacher rejection sensitivity as rejection sensitivity is linked to negative social experiences in schools (Zimmer-Gembeck et al., 2013), and experiences of discrimination as perceived discrimination has been associated with less willingness to intervene on behalf of one’s peers who are victimized (Mulvey et al., 2019).

### Bystander Intervention

Bullying can be defined as aggressive behavior which is repeated over time and which involves a power imbalance between the aggressor and the victim (Espelage and Colbert, 2016). Bullying can take many forms, including physical aggression, verbal aggression, cyberbullying, and social exclusion (Wang et al., 2010). The current study focuses on social exclusion, one type of bullying. Research indicates the powerful roles that bystanders can play in halting bullying (Mulvey et al., 2013), with results indicating that bullying tends to stop very quickly if a bystander intervenes (Hawkins et al., 2001). Bystanders have a number of different options when they observe someone else being bullied—they could defend the victim, reinforce the bully (by laughing or watching), assist the bully (participate in excluding) or distance themselves as an outsider (walk away) (Salmivalli et al., 2011). Individuals may make different decisions about how to respond depending on the type of bullying and how the bullying is occurring. Moreover, findings on bystander responses to social exclusion, one type of bullying, indicate that when bystanders observe exclusion but do not intervene, observers judge exclusion as more acceptable, suggesting the important distal impacts of bystanders (Malti et al., 2015). Given the importance of bystanders in helping to stop bullying, the focus of this study is on both moral judgments regarding one type of bullying, namely social exclusion, and factors that are associated with bystander intervention to stop social exclusion. Moral judgments are important to also consider, in addition to bystander behavior. This is because for youth to be motivated to intervene on behalf of one who is excluded, youth first need to recognize that the exclusion which is occurring is wrong. However, research suggests that adolescents often prioritize group membership (maintaining group identity) over moral principles in making decisions about if social exclusion is okay or not okay (Hitti et al., 2016).

### What Fosters an Inclusive Environment?

Our research on fostering inclusive spaces draws on the social reasoning development perspective (Rutland et al., 2010; Rutland and Killen, 2015). This perspective stems from two robust research traditions, social domain theory (Turiel, 1983) and social identity theory (Tajfel and Turner, 1976), in arguing that individuals weigh both their moral principles and sense of loyalty and identification with their groups when making social decisions. When considering inclusion and exclusion evaluations, research drawing on this perspective finds that youth consistently balance the pull of both of these concerns (Mulvey, 2016). Further, research demonstrates that youth are especially attuned to the importance of inclusion in school contexts: they judge exclusion at school (e.g., at a school dance or lunch at school) to be less acceptable than exclusion from out of school events (e.g., birthday sleepovers; Killen et al., 2010). Thus, school may be one particular context where inclusive attitudes can be fostered as there may be school norms, policies or practices that foster inclusion in schools (Nipedal et al., 2010). Moreover, with age, adolescents may place greater priority on group-based concerns than on moral principles (Killen et al., 2017). As an example, findings suggest that older adolescents are less likely to intervene when they hear peers use race-based humor at school, in part because of concerns regarding the consequences they may face for challenging their peer group (Mulvey et al., 2016).

Factors within the school environment may be centrally important for fostering inclusive tendencies. Prior research has documented the role of school norms in fostering inclusion, with findings suggesting that if children are told that their school has a norm supporting inclusion of others, they will be more likely to reject exclusion of peers, although individual group norms can also influence judgments (Nesdale, 2011; McGuire et al., 2015), but less work has examined other factors in the school environment that may shape inclusive tendencies. Further, research demonstrates that adolescents are able to articulate harm associated with exclusion experienced at school, while also recognizing the importance of maintaining group boundaries (Thorkildsen et al., 2002). This suggests that adolescents understand the complexity of exclusion. The aim of the current study is to examine specific school and teacher-related factors that may shape adolescents’ intentions to intervene to discourage social exclusion.

### School Climate

Research also demonstrates that school climate as a multidimensional construct (perceptions of dimensions of the school environment such as student–teacher relationships, social environment, differential treatment of some students, and connection to one’s school) is important for shaping moral judgments and responses to bullying, including exclusion, in school contexts (Mulvey et al., 2019). For example, beyond school norms, the school social environment more generally may be important for fostering inclusive tendencies. Students who feel happy with their peers and the overall climate for students at their school may be more likely to welcome others and be inclusive. Further, adolescents who perceived higher support from their teachers were more likely to report that they would challenge the bully and comfort the victims by being inclusive for them (Evans and Smokowski, 2015).

Research shows that students who are satisfied at school (happy and content with their school) are more likely to report that they experience positive relationships (Whitley et al., 2012). In addition to the social environment, school connectedness or school belonging is a central dimension of school climate that may shape students’ inclusive tendencies. Prior research documents positive outcomes (for instance, greater school enjoyment) for youth who feel that they are more connected to their schools or have higher school belonging ratings (Cemalcilar, 2010; Gillen-O’Neel and Fuligni, 2013). Further, research documents that belonging matters for how students think about if they might intervene if someone is being excluded: students who recognize the importance of belonging are more likely to demonstrate inclusive tendencies (Feigenberg et al., 2008).

However, there is still much that is unknown with regards to the way that students’ own feelings about their connectedness to their school or how much they belong shape their desire to include others and prevent exclusion.

Additional school climate factors such as student-teacher relationships are centrally important for shaping student attitudes and responses (Mulvey et al., 2019). Further, while we often think of student–teacher relationships as important for shaping resilience in victimized youth (Konishi and Hymel, 2009; Wang et al., 2015), recent research indicates that positive relationships with teachers are not protective for youth who experience high rates of bias-based victimization such as teasing and exclusion (Price et al., 2019). Additionally, research demonstrates that youth who report more positive student-teacher relationships are more likely to defend victims of bullying (Jungert et al., 2016). Less is known, however, about whether student-teacher relationships can foster inclusive tendencies in youth, perhaps preventing victimization from occurring.

### Teacher Rejection Sensitivity

While positive student-teacher relationships may be important, not all students have positive student–teacher relationships (McGrath and Van Bergen, 2015). In fact, some students fear rejection from their teachers (London et al., 2007) or experience exclusion and discrimination from their teachers (Benner and Graham, 2013; Respress et al., 2013; Mulvey et al., 2020). Rejection sensitivity refers to a tendency that some children hold to react defensively (either with anxiety or anger) to the potential for rejection from others in ambiguous situations (Downey et al., 1998; London et al., 2007). Findings document that youth who score higher on rejection sensitivity experience more difficulty with relationships and engage in more aggressive behavior (Downey et al., 1998; Bondü and Krahé, 2015; Zimmer-Gembeck et al., 2016; Gao et al., 2019). Further, prior work has documented that youth who feel rejected by their teachers have increasingly difficult relationships with their peers over time (Mercer and DeRosier, 2008). What has not yet been explored, however, is how teacher rejection sensitivity relates to student inclusive tendencies. It may be that adolescents who are more worried about being rejected by their teachers will be motivated to protect others from being excluded. Prior research demonstrates the importance of examining both anxious and angry subtypes of rejection sensitivity, as these subtypes are differentially related to child outcomes (Downey et al., 1998; London et al., 2007; Zimmer-Gembeck and Nesdale, 2013). Specifically, London et al. (2007) found that anxious rejection sensitivity was associated with social anxiety and withdrawal, while angry rejection sensitivity was associated with aggression. On the other hand, fears of rejection from teachers may result in adolescents’ not wanting to intervene to promote inclusion as participants may fear that actively advocating for inclusion may place them at greater risk for additional rejection from their teachers. Additionally, those who perceive that they are rejected by their teachers may not be motivated to foster others’ inclusion or may not feel that they have the capabilities to support others’ inclusion (London et al., 2007). Prior research has not previously assessed whether anxious and angry teacher rejection sensitivity are associated with expected responses to observing others’ exclusion, but we hypothesized that anxious rejection sensitivity might be more likely to be associated with responses that could promote exclusion, given its links to social anxiety and withdrawal (London et al., 2007; Zimmer-Gembeck and Nesdale, 2013) than would angry rejection sensitivity. While measures of rejection sensitivity include subscales for peer and teacher rejection sensitivity, for the current analysis we focused on teacher rejection sensitivity in order to closely examine the impact of perceptions of relationships with teachers.

### Perceptions of Discrimination

Related to rejection sensitivity, some youth may perceive that they are targeted by their teachers for discrimination or that they are excluded from opportunities provided by teachers or that others are given differential treatment (Griffin et al., 2020). Findings suggest that such perceptions of discrimination can impact one’s self-esteem (Verkuyten, 1998) and that experiences of bias and discrimination in schools are related to factors such as teacher responsiveness and multicultural education (Verkuyten and Thijs, 2002). Further, prior research has shown that perceptions of discrimination can, at times, motivate youth and emerging adults, especially those from ethnic minority backgrounds, to engage in activism to promote social change (Hope et al., 2019). Research also documents that there are different profiles of students who report high perceptions of peer and teacher discrimination with some adolescents disengaging if they experience discrimination and not expressing intentions to intervene to help others, while others increase their involvement in the bullying ecology broadly, expressing intentions to both challenge unfair treatment of others as well as to potentially participate in others’ victimization (Mulvey et al., 2020). Thus, more research is needed that examines the role of perceptions of discrimination and perceived exclusion/differential treatment in shaping youth inclusive tendencies.

### Current Study

Our focus on schools and teachers centered on factors that would encourage bystanders to defend victims of social exclusion, such as school climate (Zullig et al., 2015), as well as factors that might inhibit inclusion, such as sensitivity to being rejected by teachers (Zimmer-Gembeck et al., 2013) or perceptions of teacher or peer discrimination (Adam et al., 2015; Gutman et al., 2017). Further, in the current study, we examined 6th and 9th graders, as these grades are transition years in schools the United States (movement from elementary school to middle school and from middle school to high school) wherein peer relationships undergo significant reorganization (Farmer et al., 2013).

Our research questions were:

(1) How do student, peer and teacher factors explain adolescents’ moral judgments of exclusion?
(2) How do student and teacher factors explain adolescents’ intentions to intervene to prevent exclusion?

Our hypotheses were:

(1) Adolescents who perceive their school climate to be more positive generally (positive student–teacher relationships, greater school connectedness, lower perceived exclusion/differential treatment, and higher school social environment) would indicate more intentions to intervene to defend the victim and be less likely to respond in ways that may support the social exclusion.
(2) Adolescents who are more sensitive to rejection from their teachers, especially those who are anxious about rejection sensitivity, would be less likely to actively intervene to defend the victim and more likely to respond in ways that may promote exclusion.
(3) Adolescents who report experiencing more peer or teacher discrimination would be less likely to actively intervene to defend victims of exclusion and more likely to respond in ways that support the excluder.
(4) Consistent with prior research that documents that younger adolescents are more likely to recognize the harmful nature of exclusion (Hitti et al., 2016) and to intend to intervene to support victims (Mulvey et al., 2016), we expected that 6th graders might judge exclusion as more wrong and be more likely to expect to intervene to defend the victim than would 9th graders.
(5) Consistent with prior research documenting that girls are more likely to recognize the harmful nature of exclusion than are boys (Killen et al., 2002), we expected that girls would judge exclusion to be more wrong than would boys.

## Materials and Methods

### Participants

Our study included 896 adolescents who were 6th (*N* = 450, *M_*age*_* = 11.73, *SD* = 0.84), and 9th (*N* = 446, *M_*age*_* = 14.82, *SD* = 0.90) graders ranging between 10 and 18 years of age. Participants were approximately evenly divided by gender (49.6% of the 6th graders were female and 50.4% of the 9th graders were female) and were from five middle- to low-income public schools in the Southeastern United States. Participants were reflective of the school communities, representing primarily European-Americans (63.3%), with 22.9% African-American, 3.9% Latino, 7% Multiracial, and 2.9% other ethnic groups represented as well. The study was approved by the Institutional Review Board at the University of South Carolina. All students in the 6th and 9th grades at participating schools were invited to participate and informed opt-out consent letters were sent home to families 1 week before data was collected. Only students with parental consent who also assented to completing the study were allowed to participate (participation rate was 78%).

### Measures

#### Social Exclusion

All participants evaluated a gender-matched hypothetical bullying scenario focused on social exclusion (“Let’s say that X is ignored and left out all the time by some of X’s classmates. No one talks to X and they act like X doesn’t even exist. X does not know what to do about.”) They first completed a *moral judgment* assessment (acceptability of exclusion: How okay or not okay is it that his (her) classmates act this way? 1 = Really Not Okay to 6 = Really Okay). Then, they completed a measure of their *intervention tendencies* as a bystander: “Let’s say you thought what his classmates were doing was not okay. Pick a response for each question showing how likely or not likely you would do the following: say something to them; get help from a teacher, family member or other adults; get help from a friend; talk to the victim about it later; not get involved and stay there; or walk away” (1 = Really Not Likely to 6 = Really Likely). A factor analysis using principal components analysis was conducted on bystander responses, which indicated two factors with eigenvalues about 1. The first factor (eigenvalue = 2.72, 45.4% of variance), defending behaviors, included the responses saying something to them to get help from a teacher, family member, or other adults, get help from a friend and talk to the victim about it later (factor loadings between 0.71 and 0.83). The second factor (eigenvalue = 1.74, 23.7% of variance), non-defending behaviors, included the responses saying the individual would not get involved and stay there (0.78) and would walk away (0.72). Thus, these assessed both tendencies that would help defend the victim against exclusion (say something to them, get help from a teacher, family member or other adult; get help from a friend; and talk to the victim about it later) as well as non-defender tendencies that might further perpetuate exclusion (not get involved and stay there, and walk away).

#### Rejection Sensitivity

The Childhood Rejection Sensitivity Questionnaire (Downey et al., 1998; Zimmer-Gembeck et al., 2013) was used to measure adolescents’ rejection sensitivity. This measure included written scenarios involving peers and teachers; however, for this analysis only the teacher rejection sensitivity items were used. An example scenario was, “Now imagine that you’re back in class. Your teacher asks for a volunteer to help plan a party for your class. Lots of kids raise their hands so you wonder if the teacher will choose YOU.” Following each vignette, participants responded to three questions. The first two questions assessed anxious and angry responses by asking how nervous (e.g., “How nervous would you feel, right then, about whether or not the teacher will choose you?”; three items; α = 0.73) and how mad (e.g., “How mad would you feel, right then, about whether or not the teacher will choose you?”; three items; α = 0.76) participants would feel in the situation. Responses to these items ranged from 1 (Not Mad/nervous at all) to 6 (Very, very mad/nervous). The third question asked about the expectation of acceptance (e.g., “Do you think the teacher will choose you?”; three items; α = 0.71), with responses from 1 (YES!!) to 6 (NO!!). A separate score was created for each situation by multiplying the score for the expected likelihood of rejection by the degree of anger or anxiety over the possibility of its occurrence (expectancy of rejection X anger and expectancy of rejection X anxious) and then dividing their sum by the total number of situations (Downey et al., 1998).

#### School Climate

Participants completed the School Climate Measure (Zullig et al., 2015) which assessed perceptions of school climate on a number of dimensions using a Likert-type scale (1 = Strongly disagree to 5 = Strongly agree). The subscales of interest were: positive student-teacher relationships (eight items; example item “Students get along well with teachers”; α = 0.92), school connectedness (four items; example item “This school can make students enthusiastic about learning”; α = 0.86), perceived exclusion/differential treatment (three items; example item “At my school, the same students get chosen every time to take part in after-school or special activities; α = 0.87), and school social environment (two items; example item “I am happy with the kinds of students who go to my school”; α = 0.87).

#### Perceptions of Racial Discrimination

Self-report measures of perceptions of teacher and peer racial discrimination were used (see Wong et al., 2003; Eccles et al., 2006). The measure included two subscales, a peer/social discrimination subscale, and a teacher/classroom discrimination subscale. The peer discrimination subscale had three items that assessed perceptions of negative peer treatment due to race (e.g., getting into fights, being picked on, not being picked for teams or activities) (Likert-type: 1 = Never to 5 = Every day; α = 0.87). The teacher/classroom discrimination scale comprised five items evaluating students’ experiences of race-based discrimination in class settings by teachers in the past year (e.g., being disciplined more harshly, graded harder because of the race) (Likert-type: 1 = Never to 5 = Every day; α = 0.90).

#### Data Analytic Plan

Preliminary analyses determined that a very small amount of variance in our dependent variables was accounted for by the nesting of students within schools (intraclass correlations were 0.01–0.02). Hierarchical linear regression was used to examine predictors of participants’ moral judgments of social exclusion and their expected intervention behaviors if they observed social exclusion (see Table 1 for correlations between variables, means, and standard deviations). First, participants’ age group (dichotomous: 6th grade = 0, 9th grade = 1), ethnicity [dichotomous: ethnic majority (European-American participants) = 0, ethnic minority (non-European-American participants) = 1], and gender (male = 0, female = 1) were entered into the first step. For all intervention analyses (but not moral judgments), a dichotomous variable for participants’ moral judgment [0 = not okay (responses of 1 – 3); and 1 = okay (responses of 4 – 6) was computed and entered as the second step]. Next, teacher rejection sensitivity (angry and anxious) variables were entered into the model. In the next step, school climate (positive student–teacher relationships, school connectedness, perceived exclusion/differential treatment, school social environment) were entered and in the final step school discrimination (peer discrimination, and teacher discrimination) variables were added next. Additional regression analyses were conducted by adding interaction terms last. However, the inclusion of the interaction terms did not significantly account for the variance of outcome interest in the overall model, thus interaction terms were dropped from the final models. In order to correct for multiple comparisons, *p* < 0.005 was considered significant.

**TABLE 1:** Means, standard deviations, and correlations between variables.

**Variables**	***M* (*SD*)**	**1**	**2**	**3**	**4**	**5**	**6**	**7**	**8**	**9**	**10**
(1) Moral Judgment of Exclusion	1.77 (1.13)	−									
(2) Non-Defending Behaviors	2.51 (1.51)	0.26^*b*^	−								
(3) Defending Behaviors	4.53 (1.25)	−0.35^*b*^	−0.24^*b*^	−							
(4) Rejection Sensitivity - Anxious	10.54 (5.50)	–0.05	0.01	−0.10^*b*^	−						
(5) Rejection Sensitivity- Angry	7.99 (4.82)	0.10^*b*^	0.15^*b*^	−0.09^*b*^	0.64^*b*^	−					
(6) Positive Student-Teacher Relationships	3.59 (0.93)	−0.19^*c*^	–0.06	0.39^*b*^	–0.05	−0.18^*b*^	−				
(7) School Connectedness	3.32 (1.03)	−0.13^*c*^	0.24	0.29^*b*^	0.03	–0.07	0.70^*b*^	−			
(8) School Social Environment	3.62 (1.08)	−0.18^*c*^	–0.02	0.30^*b*^	–0.0	−0.12^*b*^	0.59^*b*^	0.63^*b*^	−		
(9) Perceived Exclusion/Differential Treatment	2.83 (1.07)	0.05	−0.08^*a*^	0.03	0.11^*b*^	0.13^*b*^	–0.01	0.08^*a*^	0.05	−	
(10) Teacher Discrimination	1.41 (0.81)	0.32^*c*^	0.26^*b*^	−0.08^*a*^	0.16^*b*^	0.33^*b*^	−0.16^*b*^	–0.03	−0.14^*b*^	0.22^*b*^	−
(11) Peer Discrimination	1.44 (0.85)	0.25^*c*^	0.23^*b*^	−0.09^*a*^	0.10^*b*^	0.25^*b*^	−0.09^*a*^	–0.01	−0.12^*b*^	0.19^*b*^	0.70^*b*^

*^*a*^p < 0.05; ^*b*^*p* < 0.01; ^*c*^*p* < 0.001.*

## Results

### Moral Judgments

For moral judgments, the final model with all variables included accounted for a significant amount of variance (15%), see Table 2. There were three significant predictors of moral judgment of social exclusion: gender (*B* = −0.26, β = −0.12, *p* = 0.001), ethnicity (*B* = −0.30, β = −0.13, *p* < 0.001), and teacher discrimination (*B* = 0.31, β = 0.22, *p* < 0.001). Further, positive student-teacher relationships approached significance (*B* = −0.12, β = −0.10, *p* = 0.04). Female and ethnic majority participants were more likely to judge the social exclusion as wrong than were male and ethnic minority participants. Further, the more teacher discrimination participants reported, the more acceptable they judged the social exclusion to be. Finally, participants with more positive student–teacher relationships were generally more likely to judge exclusion as wrong.

**TABLE 2:** Moral judgment of social exclusion.

	**Moral Judgment**		
**Variable**	***B***	***SE B***	**β**
Age	0.10	0.08	0.05
Ethnicity (Majority = 1, Minority = 0)	–0.30	0.08	−0.13***
Gender (Male = 1, Female = 0)	–0.26	0.08	−0.12***
Teacher Rejection Sensitivity-Anxious	–0.03	0.01	−0.13**
Teacher Rejection Sensitivity-Angry	0.01	0.01	0.06
Positive Student-Teacher Relationships	–0.12	0.06	−0.10*
School Connectedness	0.00	0.05	0.00
School Social Environment	–0.07	0.05	–0.06
Perceived Exclusion/Differential Treatment	–0.02	0.04	–0.02
Peer Discrimination	0.04	0.06	0.03
Teacher Discrimination	0.31	0.07	0.22
*R*^2^	0.15		
F Change	21.80***		

*p < 0.05; **p < 0.01; ***p < 0.001.

### Defender Behaviors

For bystander intervention expectations that would defend the victim of exclusion (such as confronting the excluder or talking to an adult), the final model accounted for a significant amount of variance (26%), see Table 3. There were six significant predictors of expectations to engage in behaviors that would promote inclusion if the participant observes exclusion: gender (*B* = 0.51, β = 0.20, *p* < 0.001), moral judgment (*B* = −0.88, β = −0.19, *p* < 0.001), anxious teacher rejection sensitivity(*B* = 0.04, β = 0.17, *p* < 0.001), angry teacher rejection sensitivity (*B* = −0.04, β = −0.15, *p* < 0.001), positive student–teacher relationships (*B* = 0.43, β = 0.32, *p* < 0.001), and teacher discrimination (*B* = 0.20, β = 0.13, *p* = 0.0014). School social environment (*B* = 0.12, β = 0.10, *p* = 0.012) approached significance. This revealed that female participants, those who were more anxious about being rejected by their teachers, those with more positive student-teacher relationships and those who report more teacher discrimination were more likely to expect that they would intervene to defend the victim. Additionally, those who experience more positive school social environments were generally more likely to expect that they would intervene. However, participants who judged the exclusion to be acceptable and those who were more angry about teacher rejection were less likely to expect that they would intervene to defend the victim.

**TABLE 3:** Bystander intervention in response to social exclusion: defending behaviors.

	**Defending Behaviors**		
**Variable**	***B***	***SE B***	**β**
Age	0.04	0.08	0.02
Ethnicity (Majority = 0, Minority = 1)	0.07	0.08	0.03
Gender (Male = 1, Female = 0)	0.51	0.08	0.20***
Moral Judgment (0 = not okay; 1 = okay)	–0.88	0.15	−0.19***
Teacher Rejection Sensitivity-Anxious	0.04	0.01	0.17***
Teacher Rejection Sensitivity-Angry	–0.04	0.01	−0.15***
Positive Student-Teacher Relationships	0.43	0.06	0.32***
School Connectedness	–0.02	0.06	–0.02
School Social Environment	0.12	0.05	0.10*
Perceived Exclusion/Differential Treatment	0.04	0.04	0.03
Peer Discrimination	–0.07	0.06	–0.04
Teacher Discrimination	0.20	0.07	0.13**
*R*^2^	0.26		
F Change	4.42**		

*p < 0.05; **p < 0.01; *** *p* < 0.001.

### Non-defender Behaviors

For bystander intervention expectations of engaging in behaviors that would not defend the victim and might promote exclusion, such as walking away or not taking any action, the last model with all variables included, accounted for a significant amount of variance (11%), see Table 4. There were five predictors of expectations of promoting exclusion that were significant or that approached significance: moral judgment (*B* = 0.40, β = 0.07, *p* = 0.04), anxious teacher rejection sensitivity (*B* = −0.03, β = −0.10, *p* = 0.01), angry teacher rejection sensitivity (*B* = 0.05, β = 0.14, *p* < 0.001), perceived exclusion/differential treatment (*B* = 0.13, β = 0.1, *p* = 0.007) and teacher discrimination (*B* = 0.20, β = 0.11, *p* = 0.034). This revealed that those who were more anxious about being rejected by their teachers were less likely to engage in non-defender behaviors. However, participants who judged the exclusion to be acceptable, those who reported that they were more angry about teacher rejection, those who perceived more differential treatment at school and those who report experiencing more teacher discrimination were more likely to report that they would engage in non-defender behaviors.

**TABLE 4:** Responses to social exclusion: non-defending behaviors.

	**Non-defending Behaviors**		
**Variable**	***B***	***SE B***	**β**
Age	0.14	0.11	0.05
Ethnicity (Majority = 0, Minority = 1)	–0.16	0.11	–0.05
Gender (Male = 1, Female = 0)	–0.19	0.10	–0.06
Moral Judgment (0 = not okay; 1 = okay)	0.40	0.19	0.07*
Teacher Rejection Sensitivity-Anxious	–0.03	0.01	−0.10**
Teacher Rejection Sensitivity-Angry	0.05	0.01	0.14***
Positive Student-Teacher Relationships	0.05	0.08	0.03
School Connectedness	0.07	0.07	–0.05
School Social Environment	–0.07	0.06	–0.05
Perceived Exclusion/Differential Treatment	–0.13	0.05	0.10**
Peer Discrimination	0.15	0.08	0.08
Teacher Discrimination	0.20	0.09	0.11*
*R*^2^	0.11		
F Change	9.95***		

*p < 0.05; **p < 0.01; ***p < 0.001.

## Discussion

Our novel results revealed the importance of school climate, teacher rejection sensitivity and perceptions of discrimination for promoting inclusive tendencies. We also documented intriguing differences based on participant demographics, including gender and ethnicity. Results indicated that girls and participants who perceived better relationships between students and teachers were more likely to judge exclusion to be wrong. Further, ethnic minority participants, those who were more anxious about being rejected by their teachers and those who reported more teacher discrimination were less likely to judge the exclusion as wrong. In general, participants who recognized the harmful nature of exclusion, and those who reported more positive student-teacher relationships, and who perceived a more positive school social environment were more likely to expect that they would defend victims against exclusion. On the other hand, participants who reported being more angry about teacher rejection, who believed that some students received differential treatment at school, and those who saw the exclusion are more okay were more likely to expect they would respond in ways that would not defend victims of exclusion such as saying nothing.

### School Climate

Our results documented the complex interplay of school and teacher factors in shaping adolescents’ inclusive tendencies. In terms of school climate, we find that positive student–teacher relationships are of central importance in defending behaviors: the more positive adolescents’ report their relationships with their teachers to be, the more wrong they recognize exclusion to be. Further, recognizing exclusion as wrong is a critical foundation for intervention: youth who report that exclusion is wrong and those who report more positive student–teacher relationships are more likely to engage in behaviors that will encourage inclusion such as to speak up, to get help from peers and adults, and to talk to the victim when someone is excluded. Interestingly, school connectedness was not a key factor in accounting for inclusive behavior. This may be because the particular school connectedness items used in this measure capture teachers creating positive learning environments (exciting coursework, enthusiasm around learning, feeling as though teachers take student feedback on possible courses) (Zullig et al., 2015). In line with this, school social environment, which captures more completely belonging with peers, is positively related to behaviors that will encourage inclusion. Finally, perceived exclusion/differential treatment, which captures feeling as though some students are denied opportunities that others are afforded at school (“the same person always gets to help the teacher”), positively predicts behaviors that might promote exclusion, such as not getting involved. This suggests that adolescents who perceive that their school fosters differential treatment of some students may disengage and not seek out opportunities to help others who they observe being excluded. These findings highlight the nuanced way in which different elements of the school climate shape adolescents’ inclusive orientation. Teacher factors, perceptions of the environment at school, and peer factors can all play a role in how adolescents think about and respond to the exclusion of others.

### Teacher Rejection Sensitivity

Interestingly, the findings also suggest that youth who are sensitive to being rejected by their teachers also respond differently to exclusion. Contrary to our hypotheses, when adolescents are more anxious about being rejected by their teachers, they judge exclusion unacceptable, and seek to defend victims of exclusion. This suggests that anxious rejection sensitive youth are attuned to the harmful nature of, and are willing to help prevent, exclusion. We expected that anxious rejection sensitive youth might disengage and not want to help others because they may be anxious about further rejection. However, we find that they are actually engaged in defending behaviors. Thus, these students may be attending more to preventing rejection of others as opposed to concerned with experiencing additional rejection themselves. Future research might further explore this with qualitative interviews with students who are rejection sensitive to more completely understand their decision-making when they observe others’ exclusion. Importantly, though, participants who were angry about possible rejection from their teachers look quite different: angry youth were less likely to defend the victim. Prior research demonstrates that rejection sensitive youth may engage in higher rates of aggression (Webb, 2008; Bondü and Krahé, 2015), and that rejection sensitive youth are more likely to not intervene if they observe aggression (Gönültaş et al., 2019). These findings extend this prior work (London et al., 2007; Zimmer-Gembeck and Nesdale, 2013) by demonstrating differential patterns for youth who are anxious and angry about possible teacher rejection.

### Perceptions of Discrimination

Our findings suggest that a positive school climate can promote intentions to intervene. Surprisingly, our results also demonstrate that participants who perceive that their teachers discriminate against them were more likely to indicate that they would both promote and discourage inclusion. It may be that these youth do not want to be present when others are excluded, and thus seek opportunities to avoid the situation or to seek out help away from the instance of exclusion. It could be that they want to disengage from the immediate instance of exclusion because they fear being falsely accused of being involved, given that they report prior experiences of teacher discrimination. Perhaps they are concerned that their attempts to intervene directly would be misinterpreted and that they would be seen as culpable. Given this pattern of teacher discrimination being associated with both wanting to promote and challenge exclusion, future research may need to explore more carefully specific intervention behaviors to uncover if these findings are being driven by particular behaviors. Finally, although there were no differences in how ethnic minority and ethnic majority participants expected they would respond if they observed social exclusion occurring, ethnic minority adolescents judged the act of social exclusion as less acceptable than did ethnic majority youth. This is important as prior research indicates that ethnic minority youth who experienced discrimination can be motivated to engage in civic activism (Hope et al., 2019). Our findings suggest that ethnic minority peers may be especially attuned to how harmful exclusion can be. Interestingly, although they are more likely to recognize that exclusion is harmful, this does not translate into increased intentions to defend those who are excluded. Future research should aim to identify additional factors that may promote intentions to intervene.

The set of findings suggest the importance of examining predictors of upstander behavior for ethnic minority youth and those who perceive that they are the victims of discrimination. Prior research documents that there is heterogeneity in responses to peer aggression in youth who perceive that they are the victims of discrimination, with some youth motivated to challenge other aggressors, while others even become involved in bullying others (Mulvey et al., 2020). Thus, more work is needed to understand how to shift cognitive patterns and empower youth who experience discrimination or are marginalized to harness their experiences to help others by fostering inclusion. Further, additional research should explore whether having peers who share your experiences (for instance, ethnic identity) or having a stronger sense of ethnic identity (Mathews et al., 2019) may propel youth toward fostering inclusion for others.

### Gender and Age Differences

Further, we document age and gender findings. Interestingly, while much prior research has documented that younger adolescents are more likely to engage in bystander intervention (Mulvey et al., 2016, 2019), in this study, 6th and 9th-grade adolescents did not differ in their judgments or expected responses. This is important as it suggests that older adolescents, may, at times, be just as likely as younger adolescents to recognize how harmful exclusion is, even though prior research finds that adolescents are often more accepting of exclusion with age (Hitti et al., 2016). In terms of gender, we find that girls are more likely to judge the exclusion wrong, and to expect that they will respond in ways that defend the victim. These findings are consistent with prior research that documents that girls are often acutely attuned to the harmful nature of exclusion (Killen et al., 2002). The findings also suggest the importance of encouraging inclusive behavior not only among girls, but also among boys. Stereotypes often suggest that girls are more likely to engage in relational aggression, such as exclusion (Crick and Grotpeter, 1995), even though recent findings suggest that relational aggression is equally common among boys and girls (Lansford et al., 2012). These stereotypes, however, may lead to boys and girls being socialized differently around issues of social exclusion, with girls more likely to be encouraged to stop exclusion and engage in inclusive practices as a result of misperceptions about girls having a higher likelihood of excluding others. Thus, future research might involve qualitative interviews with boys and girls about how the messages they hear about exclusion can be used to identify if boys and girls are encouraged to be inclusive in similar ways.

### Limitations and Future Directions

While this work provides important insight into how schools can foster inclusive tendencies, it does have some limitations. First, the focus of this research was on adolescents, yet children report experiencing exclusion well before adolescence (Elenbaas and Killen, 2016), which suggests that it may be important to identify factors that foster inclusion in children as well to have a more comprehensive developmental story. Further, this research was cross-sectional, which is helpful in identifying critical factors that may be important targets for intervention. However, it will be important for future research to examine longitudinal and bi-directional relationships between school and teacher factors, as well as one’s own experiences of exclusion and youth attitudes toward exclusion. Longitudinal research will be able to also identify possible causal factors, which the current study cannot. Finally, the current study includes assessment of hypothetical scenarios. While expected behaviors reported in response to hypothetical scenarios align well with reports of actual behavior (Turiel, 2008), it will still be important for future research to gather data using multiple sources of information such as teacher reports of inclusive behavior or peer nominations of which students do intervene to stop exclusion. It may also be helpful to examine family relationship quality and other environmental contexts such as neighborhood safety that may contribute to perceptions and inclusive tendencies.

## Conclusion and Implications

The novel findings in our study document that school climate can shape adolescents’ attitudes toward inclusion. Further, findings highlight places for intervention. Youth who are sensitive to possible rejection from their teachers and who perceive that they have been discriminated against by peers or teachers are less likely to defend victims of exclusion. Thus, school programming to foster inclusion should work to ensure that students feel welcomed and included and seek to root out instances of discrimination or differential treatment in order to foster inclusion. Additionally, interventions might aim to target youth cognition to increase bystanders’ motivation to intervene in situations involving social exclusion—helping to instruct how to accurately interpret social cues from both peers and teachers (Arsenio and Lemerise, 2004) — to create environments conducive to inclusive behavior. The implications of this work suggest the importance of school-wide approaches to creating inclusive climates with attention to climate, peer relationships, student–teacher relationships, and student experiences. Additionally, the findings suggest the importance of recognizing the harmful nature of exclusion. Parents, and teachers can work to foster discussions with students about the importance of inclusion. In sum, our results suggest that generally youth recognize the harmful nature of exclusion and are willing to intervene if they observe others being excluded.

## Data Availability Statement

The raw data supporting the conclusions of this article will be made available by the authors, without undue reservation.

## Ethics Statement

The studies involving human participants were reviewed and approved by the University of South Carolina IRB with inter-institutional agreement by the North Carolina State University IRB. Written informed consent for participation was not provided by the participants’ legal guardians/next of kin because: we used written informed consent, using a passive or opt-out consent form (parents responded if they did not want their child to participate). This was the informed consent procedure approved by our IRB and the school district.

## Author Contributions

KM, MI, CD, and RC designed the study. KM, SG, and GI collected the data for the study. KM and SG conducted the analyses. KM drafted the manuscript. SG, GI, RC, CD, and MI edited and reviewed the manuscript. All the authors contributed to the article and approved the submitted version.

## Conflict of Interest

The authors declare that the research was conducted in the absence of any commercial or financial relationships that could be construed as a potential conflict of interest.
